# Editorial: Application of multi-omics analysis in gametogenesis and the relevant human infertility diseases

**DOI:** 10.3389/fendo.2023.1229992

**Published:** 2023-06-14

**Authors:** Huan Zhang

**Affiliations:** Division of Reproduction and Genetics, First Affiliated Hospital of USTC, Hefei National Research Center for Physical Sciences at the Microscale, the CAS Key Laboratory of Innate Immunity and Chronic Disease, School of Basic Medical Sciences, Division of Life Sciences and Medicine, Biomedical Sciences and Health Laboratory of Anhui Province, University of Science and Technology of China, Hefei, China

**Keywords:** multi-omics, gametogenesis, infertility, animal models, infertile patients

Germ cells transmit genetic material from parents to offspring. Gametogenesis includes spermatogenesis and oogenesis, which undergo regulated processes of mitosis, meiosis and maturation. Abnormal gametogenesis can cause infertility, affecting 12-15% of reproductive-age couples (1). Genetic defects cause nearly 50%, with environmental factors exacerbating the issue. With the rapid development of various high-throughput omics technologies, Research Topics on the molecular basis of gametogenesis and the pathogenesis of the relevant human infertility diseases have become increasingly abundant. Studies with single omics only provide partial insight into the underlying mechanism, while multi-omics joint analysis is necessary for understanding interactions among DNA, RNA, proteins, and metabolites for insights into gametogenesis and infertility. We are pleased to present a Research Topic of five articles that explore the latest research in this field (Chen et al., Liu et al., Shi et al., Zeng et al., Zhang et al.). These articles provide a comprehensive overview of the latest research in the application of multi-omics analysis in gametogenesis and infertility.

In spermatogenesis, Shi et al. integrated multiple omics data to explain the unique transcriptional regulatory network of spermatogonial stem cells (SSCs) and identified the hub SSC-specific genes and key SSC-specific transcription factors. Chen et al. studied the role of CEP72, a critical component of the centrosome in male fertility. They found that although the *Cep72* knockout male mice were fertile, their sperm exhibited abnormal flagellum structures. Through transcriptome analysis, several genes were identified to be related to sperm morphogenesis. Besides the studies focus on spermatogenesis in the testis, Liu et al. conducted combined omics analysis and immunofluorescent labelling to demonstrate that epididymal epithelial degeneration and lipid metabolism impairment contribute to male infertility in *Ocln* knockout mice. In oogenesis, Zeng et al. identified a homozygous nonsense mutation in *ZP2* in a female infertile patient, and studied the pathogenicity of the mutation using transcriptome sequencing of *Zp2* mutant rat oocytes. Furthermore, Zhang et al. used single-cell RNA sequencing (scRNA-seq) to compare the characteristics of peripheral blood mononuclear cells (PBMC) from patients with Premature Ovarian Insufficiency (POI) and explored the potential involvement of immune response in idiopathic POI.

Taken together, we can summarize the following points:

1. Application of multi-omics analysis in reproductive biology: All five articles utilized various high-throughput technologies, such as whole exome sequencing, transcriptomics, proteomics, and epigenomics, to investigate the molecular mechanisms underlying gametogenesis and infertility.

2. Identification of key regulatory factors: The articles identified several key regulatory factors, such as critical components of the centrosome, key regulators, and transcription factors that play important roles in gametogenesis and infertility.

3. Potential therapeutic targets: The articles proposed potential targets for therapeutic intervention, which could be used to improve the efficiency of *in vitro* SSC induction or treat infertility; *OCLN* and *ZP2*, which could be used as molecular diagnostic markers for related male or female infertility, respectively.

4. Limitations and future directions: While multi-omics analysis has provided valuable insights into the molecular mechanisms underlying gametogenesis and infertility, there are still limitations and challenges that need to be addressed. For example, the complexity of the data generated by multi-omics analysis requires sophisticated bioinformatics tools for data integration and analysis. In the future, research in this field will likely focus on developing more advanced technologies and analytical methods to overcome these challenges and deepen our understanding of reproductive biology.

Overall, the articles in this Research Topic demonstrate the power of multi-omics analysis in advancing our understanding of gametogenesis and infertility, and provide a foundation for future research in this field. We hope that this Research Topic will inspire further research in this field and ultimately lead to new therapies for infertility and other reproductive disorders.

## Author contributions

The author confirms being the sole contributor of this work and has approved it for publication.
